# The impact of immigration detention on mental health: a systematic review

**DOI:** 10.1186/s12888-018-1945-y

**Published:** 2018-12-06

**Authors:** M. von Werthern, K. Robjant, Z. Chui, R. Schon, L. Ottisova, C. Mason, C. Katona

**Affiliations:** 1 Helen Bamber Foundation, Bruges Place, 15-20 Baynes Street, London, NW1 0TF UK; 2 Health Services and Population Research Department, Kings College London, London, UK; 3 Division of Psychiatry, University College London, London, UK

**Keywords:** Mental health, Vulnerability, Immigration detention, Asylum seekers, Refugees

## Abstract

**Background:**

The number of asylum seekers, refugees and internally displaced people worldwide has increased dramatically over the past 5 years. Many countries are continuing to resort to detaining asylum seekers and other migrants, despite concerns that this may be harmful. In light of the considerable body of recent research, this review aims to update and expand on a 2009 systematic review on the mental health consequences of detention on adult, adolescent and child immigration detainees, which found (on the basis on 9 studies) that there was consistent evidence that immigration detention had adverse effects on mental health.

**Methods:**

Three databases were searched using key terms relating to immigration detention and mental health. Electronic searches were supplemented by reference screening. Studies were included if they were quantitative, included individuals detained for immigration purposes, reported on mental health problems and were published in peer-reviewed journals. Two reviewers independently screened papers for eligibility, and a further two reviewers completed quality appraisals for included studies.

**Results:**

Twenty- six studies (21 of which were not included in the 2009 review) reporting on a total of 2099 participants were included in the review. Overall, these studies indicated that adults, adolescents and children experienced high levels of mental health problems. Anxiety, depression and post-traumatic stress disorder were most commonly reported both during and following detention. Higher symptom scores were found in detained compared to non-detained refugees. In addition (and more clearly than was evident in 2009), detention duration was positively associated with severity of mental symptoms. Greater trauma exposure prior to detention was also associated with symptom severity.

**Conclusions:**

The literature base reviewed in this paper consistently demonstrated severe mental health consequences amongst detainees across a wide range of settings and jurisdictions. There is a pressing need for the proper consideration of mental health and consequent risk of detention-related harm in decisions surrounding detention as well as for improved care for individuals within detention facilities. Recommendations based on these findings are presented, including increased focus on the identification of vulnerability and on minimising the duration of detention.

**Electronic supplementary material:**

The online version of this article (10.1186/s12888-018-1945-y) contains supplementary material, which is available to authorized users.

## Background

The number of people forcibly displaced worldwide as a result of persecution, conflict, generalized violence or human rights violations has increased dramatically in the last 5 years. The UNHCR estimates that there are currently (July 2018) 68.5 million forced migrants around the world [1].

Many countries detain asylum seekers in immigration detention centres whilst their applications are processed or following a decision to refuse them protection. At present the total number of third-country nationals held in immigration detention in the European Union is estimated to be 158,792 [2]. In the UK alone, this figure is 32526 people, representing 20% of total EU detainees, despite a much smaller percentage of asylum applications.

There is robust and consistent evidence that refugees are more vulnerable to mental illness, particularly depression and PTSD, as compared to the general population [3, 4]. Recent evidence indicates that vulnerability to non-affective psychoses is also increased in refugees compared to other migrants, reflecting their severe and often repeated exposure to adversity [5]. In addition to pre-migration factors such as exposure to torture or human trafficking, post-migration factors, including prolonged asylum procedures, prohibition from working, poverty and poor housing are significantly associated with poor mental health [6–9]. Research from the UK also indicates that post-migration and asylum-related stressors are associated with PTSD symptoms and emotional distress in asylum seekers and refugees [10].

Time spent in immigration detention in the host country is a particular post-migration stressor that entails loss of liberty and the threat of forced return to the country of origin. For many asylum seekers with a history of major trauma, it is reminiscent of contexts in their country of origin where they had been deprived of their liberty and human rights [11]. Immigration detention also exposes asylum seekers to possible abuse from staff and violence from fellow detainees, social isolation, and forceful removal. In the UK, the Medical Justice charity has documented these problems in a series of reports [12].

The practice of detaining asylum seekers, a group with a pre-existing vulnerability to mental health problems due to higher exposure to trauma pre- and peri-migration, [4] risks further exacerbating their mental health difficulties. The experience of detention may act as a new stressor, which adds to the cumulative effect of exposure to trauma, leading to an increased likelihood of developing mental health difficulties such as PTSD as a result of the ‘building block effect’ [13]. Indeed, a 2009 systematic review reporting on the effects of immigration detention on mental health found detainees to have high levels of anxiety, depression and post-traumatic stress disorder [14]. Suicidal ideation and deliberate self-harm were also common. Severity of distress was significantly greater in those who had been detained for relatively long periods [14]. The studies reviewed outlined the extent and severity of the mental health problems observed in detained people. The authors noted this research area was still in its infancy and highlighted the urgent need for further research on both the acute and longer-term impact of immigration detention on mental health, as well as its independence or otherwise from other risk factors.

Since 2009, a number of new studies have been published in the context of rising levels of forced migration globally and the frequent use of immigration detention to manage the increasing numbers of asylum seekers.

These findings have led to increasing concern that immigration detention may constitute a significant component of the post-migration adversity experienced by some asylum seekers. Such concerns over the treatment, support and management of both children and adults within detention facilities have been continuously expressed by numerous institutions across several countries, with many calling for legislative amendments to end immigration detention, particularly of children [15–17]. More recently, the UNHCR has also voiced concern over the physical and psychological effects of possible mandatory detention of all asylum seekers in Hungary [18]. The European Commission have reviewed the (very variable) use of alternatives to detention across the European Community. [19]

There has also been very extensive recent concern at the potential adverse consequences and human rights implications of the separation of migrant children from their parents by the USA authorities [20]. This led to this practice being discontinued [21].

In 2002, Australia was unique in its policy of indefinite, non-reviewable, mandatory detention of asylum seekers without valid entry documents arriving by boat. Clinical observation raised initial concerns, followed by the publication of research demonstrating the high prevalence of mental disorder in detention [22, 23]. Whereas the Australian authorities have since made changes to the law [24] that could be considered progress, the British government have sought to expand the capacity of immigration detention centres [25].

Although concerns have been raised over the mental health implications of immigration detention globally [26], we have taken the UK as a more in-depth example due to our familiarity with it and to the relatively large number of people detained there, incongruent with the comparatively small total number of asylum applications.

In 2015 a cross-party inquiry found that the UK detains too many people and for too long, and is more costly and less efficient than alternative systems operating in countries such as Australia and Sweden that have introduced community-based programmes to monitor asylum seekers and irregular migrants [27]. UK policy developments, therefore, provide an indicative illustration of these developing concerns.

In 2013, the Royal College of Psychiatrists issued a position statement on the detention of people with mental disorders in Immigration Removal Centres, which concluded that ‘detention centres are likely to precipitate a significant deterioration of mental health in the majority of cases, greatly increasing both the suffering of the individual and the risk of suicide and self-harm’ and therefore that ‘people with mental disorder should only be subjected to immigration detention in very exceptional circumstances’ [28].

In 2016, a government-commissioned review on the welfare of immigration detainees [29] highlighted the ‘evident ethical, policy and practical implications’ of medical research showing that immigration detention itself can seriously damage the mental health of detainees. A narrative review included within the report highlighted the particular vulnerability of people with a history of trauma and those with pre-existing mental or physical health problems to the adverse mental health effects of detention [30].

The UK Home Office published a Policy Guidance document on adults at risk in immigration detention [31]. which identified several ‘conditions or experiences which will indicate that a person may be particularly vulnerable to harm in detention’. ‘Immigration factors’ enabled decisions to detain to be made and maintained despite substantial vulnerability factors being present. The Royal College of Psychiatrists issued a Position Statement expressing concern that the definition of torture within the new Adults at Risk policy added ‘unnecessary and inappropriate complexity that does not assist in identifying those who are particularly vulnerable to the adverse effects of detention’ [32]. In response to legal challenge, The UK Home Office has since changed this definition [33].

This review was conducted to provide a fuller and more up-to-date synthesis of the evidence. Its specific aims were to document (a) the prevalence and types of mental health problems among child and adult immigration detainees and (b) risk factors associated with mental health problems among immigration detainees. We looked particularly for evidence about the possible effects of pre-existing trauma and/or mental health problems and of duration of detention. Since detention systems vary between countries and nomenclature surrounding detainees vary across studies, the review takes a broad and inclusive stance and includes material on people detained within an immigration context (spanning asylum seekers, refugees and other migrants).

## Methods

A systematic literature review following a modified version of the PRISMA guidelines [34] was conducted between October 2015 and May 2018 (PROSPERO Registration No: CRD42017056444).

### Inclusion and exclusion criteria

Studies were included if they (a) included male or female adults or children detained for immigration purposes; (b) reported the prevalence of mental health problems; and (c) presented results of published peer-reviewed research based on one or more of the following study designs: cross-sectional survey; case–control study; cohort study; case series analysis; experimental study with baseline measures for the outcomes of interest; or secondary analysis of organisational records (Table 1). No restrictions were placed on country setting, country of origin of the immigration detainees, or method of measuring mental health outcomes. Since research in this area remains scarce, studies were not excluded on the grounds of their quality (though this is reported in Table 2). Qualitative studies will be presented in a subsequent paper.

**Table 1:** Mental Health Problems in Immigration Detainees: Characteristics of 26 quantitative studies

Author and year	Study Design	Sample	Country of Origin	Country of Study	Outcomes of interest	Method of assessing outcome
Brabeck & Xu [59]	Cross sectional survey	*N* = 132 immigrant parents who accessed immigrant community organisations Detention Duration: unknown	Various Latin American countries	United States	Impact of detention / deportation on Latino immigrant families	Self-report survey informed by Brabeck et al. [59] Translated and back translated
Cleveland & Rousseau [38]	Cross-sectional, comparison survey	*N* = 122 detained adult asylum seekers, *N* = 66 non-detained adult asylum seekers Detention Duration: mean 31.2 days, median 17.5 days	Unknown	Canada	Anxiety, depression & PTSD	HTQ, HSCL-25, modified DEC
Coffey et al. [40]	Cross-sectional semi-structured interview and survey	*N* = 17 refugees Detention Duration: mean 3 years 2 months, range 1.6 – 4.5 years	Afghanistan, Iraq, Iran and neighbouring countries	Australia	Experience of detention PTSD, depression, anxiety, quality of life	Semi-structured interviews; HSCL-25, HTQ, WHOQOL-Brief
Cohen [50]	Data comparison	*N* = 231 self-harm incident reports *N* = 12 suicide incident reports from IRCs *N* = 38 suicides from coroners and ombudsman reports	Unknown	UK	Rates of self-harm and suicide	Analysis of records, coroner’s files and ombudsman’s reports
Cwikel et al. [37]	Cross-sectional, comparison survey	*N* = 47 female brothel workers in detention, *N* = 55 female brothel workers Detention Duration: unknown	Former Soviet Union	Israel	PTSD, depression, somatic symptoms, suicidality	PCL, CES-D, constructed scale of physical symptoms, constructed scale of both past trauma and work trauma Russian speaking psychiatrist
Graf et al. [47]	Cross-sectional survey	*N* = 80 males detained for violation of the Swiss Aliens Act Detention Duration: 4 days	31 different countries	Switzerland	Prevalence rates of mental health disorders	BJMHS, CIDI (clinical psychologist), SCL-90R, subjective mental health questionnaire within 4 days of detention, SCL-90R and self-report again 6 months later where possible Material professionally translated into several languages
Green & Eager [36]	Cross-sectional analysis of health records	*N* = 720 Detention Duration: range 3 – 24 months	58 different countries	Australia	Estimated incidence rates of new health conditions, new mental health conditions and new injuries for each cohort	Health records coded by clinical coder
Hedrick [49]	Analysis of incident reports	Unknown	Unknown	Australia	Self-harm	Self-harm incident reports
Ichikawa et al. [41]	Cross-sectional, comparison survey	*N* = 18 former detained asylum seekers, *N* = 37 non-detained asylum seekers Accessible through a group of voluntary lawyers representing them and two NGO’s Detention Duration: median 7 months, range 4 – 10 months	Afghanistan	Japan	Anxiety, depression, PTSD	HSCL-25, HTQ Translated into Dari and read out to participants by NGO caseworkers
Keller, Rosenfeld et al. [44]	Cross-sectional survey	*N* = 70 detained asylum seekers Detention Duration: median 7 months, range 2 – 42 months	Multiple countries	USA	Anxiety, depression, PTSD	HSCL-25, HTQ (+ two month follow up) Scales translated by interpreter, interviewed by experienced physician
Lorek et al. [52]	Cross-sectional survey	*N* = 11 detained children, *N* = 9 parents who responded to free legal assistance Detention Duration: range 11 – 115 days	Multiple	Britain	Mental and physical health of children held within immigration detention centre	Clinical diagnostic interviews, SCAS, DSRS, R-IES-13, SDQ, CORE, observations Psychologist and paediatricians had all been trained in carrying out cross-cultural assessments and worked regularly with asylum seeking children and families
Mares [54]	Secondary analysis of Australian Human Rights Commissions Data Set	*N* = 131 adults and *N* = 35 completed K10 N = 70 completed SDQ Detention Duration: mean 8 months, range 90-390 days	Unknown	Australia	Psychological distress, behavioural difficulties	K10, SDQ
Mares & Jureidini [53]	Assessment of referrals into CAMHS	*N* = 16 adults, 20 children, 10 families Detention Duration: mean 1 year 3 months, range 12 – 18 months	Iran, Iraq, Afghanistan, Palestine	Australia	Clinical assessment	Clinical instrument unknown
Momartin et al. [51]	Cross-sectional survey	*N* = 49 former immigration detainees on temporary protection visas and *N* = 67 granted permanent protection visas Detention duration: mean 12 months	Unknown	Australia	PTSD, anxiety, depression, general health, living difficulties and experiences of detention	HTQ, HSCL-25, GHQ-30, MOSSF-12, PMDC, DEC
Puthoopparambil et al. [46]	Cross-sectional survey	*N* = 127 immigration detainees Detention Duration: mean 37.8 days	46 different countries	Sweden	Quality of life	WHOQOL-BREF – incl. Six psychological questions Authorised telephone interpreters (used by 77 participants)
Robjant et al. [11]	Cross-sectional, comparison survey	N = 67 detained asylum seekers, *N* = 30 detained former prisoners, N = 49 asylum seekers living in the community Detention Duration: unknown	Unknown	UK	PTSD, depression, anxiety	HADS, IES-R, PDS English
Rojas-Flores [57]	Cross sectional comparison survey	*N* = 39 children of detained or deported parents *N* = 42 unauthorised no history of detention or deportation N = 16 Legal permanent resident Detention Duration: unknown	Mexico or Central America	US	PTSD, depression, behavioural difficulties, daily functioning	UCLA PTSD-RI, CES-DSC, BASC-2 PRS-C, TSCYC-SP, BASC-2 TRS-C, CAFAS
Rothe et al. (2002a)	Cross sectional survey	*N* = 74 adolescents Detention Duration: 4-6 months	Cuba	US	PTSD, psychological distress	PTSDRI, checklist of PTSD symptoms
Rothe et al. (2002b)	Cross sectional survey	*N* = 87 adolescents Detention Duration: mean 6-8 months	Cuba	US	PTSD, behavioural difficulties	PTSDRI, CBCL-TRF
Sen et al. [45]	Observational, Cross-sectional	*N* = 101 male detained in immigration removal centre Detention Duration: unknown	27 different countries	UK	Neurodevelopmental	MINI v6, SAPAS, AQ-10, ASRS, LDSQ, CANFOR No interpreters
Sobhanian et al. [42]	Cross-sectional survery	*N* = 150 former refugee detainees Detention Duration: mean 11.3 months, range 2 – 21 months	Iran, Afghanistan	Australia	Psychological status and quality of life	T-FAST, QOLI, POMS, SIS Translated and back translated into Farsi, self report administered under supervision of clinical psychologist
Steel et al. [23]	Cross-sectional	N = 10 families (14 adults, 20 children) held for in immigration detention for more than two years Detention Duration: mean 2 years 4 months, range 2 years 2 months – 2 years 8 months	Unknown	Australia	Psychiatric Status	DEC, DSC, K-SADS-PL, SCID-IV, Parenting Questionnaire, Over the telephone by same language-speaking psychologists with prior professional experience working with refugees
Steel et al. [43]	Cross-sectional, comparison survey	*N* = 241 Arabic-speaking Mandaean refugees Detention Duration: median 6 months	Mainly Iran and Iraq	Australia	Anxiety, depression & PTSD	HTQ, HSCL-25, MOSSF-12, PMLD, DEC, DSC Translated and back translated by certified Arabic-speaking healthcare interpreter
Steel et al. [39]	Longitudinal survey	N = 47 former immigration detainees on temporary protection visas and *N* = 57 granted permanent protection visas attending a state-wide early intervention program in New South Wales Detention Duration: meadian 8 months, range 1 – 30 months	Iran, Afghanistan	Australia	Anxiety, depression and PTSD	HTQ, HSCL-25, GHQ-30, PSWQ, PMLD Baseline and 2-year follow up Measures translated by experienced clinical psychologist fluent in both dialects, and back translated. Interviews undertaken by Fasi / Dari speaking psychologists
Young & Gordon [48]	Secondary analysis of Australian Human Rights Commissions Data Set	*N* = 1354 detained refugees, asylum seekers and ‘others’	Unknown	Australia	PTSD, mental health	K10, HTQ, HoNOS, HoNOSCA
Zwi et al. [58]	Cross sectional survey	*N* = 48 detained children N = 38 child asylum seekers in community	Eastern Mediterranean, South East Asia, Pacific, Africa, Stateless origin	Australia	Behavioural difficulties	SDQ

*ASRS* Adult ADHD Self-Report Scales, *AQ-10* Autism-Spectrum Quotient 10, *BASC-2 PRS-C* Behaviour Assessment System for Children-2nd Edition, Parent Rating Scales-Child, *BASC-2 TRS-C* Behaviour Assessment System for Children-2nd Edition, Teacher Rating Scales-Child, *BJMHS* Brief Jail Mental Health Screen, *CAFAS* Child and Adolescent Functional Assessment Scale, *CAMHS* Child and Adolescent Mental Health Services, *CANFOR* Camberwell Assessment of Needs – Forensic Version, *CBCL-TRF* Child Behavioral Check List – Teachers Report Form, *CES-D* Center for Epidemiologic Studies Depression, *CES-DSC* Centre for Epidemiologic Studies Depression Scale for Children, *CIDI* Composite International Diagnostic Interview, *CORE* Clinical Outcomes in Routine Evaluation, *DEC* Detention Experience Checklist, *DSC* Detention Symptom Checklist, *DSRS* Birleson Depression Self-Rating Scale, *GHQ-30* General Health Questionnaire 30, *HADS* Hospital Anxiety Depression Scale, *HSCL-25* Hopkins Symptoms Checklist 25, *HoNOS* Health of Nation Outcomes Scale, *HoNOSCA* Health of Nation Outcomes Scale for Children and Adolescents, *HTQ* Harvard Trauma Questionnaire, *IES-R* Impact of Events Scale Revised, *K10* Kessler 10, *K-SADS-PL* Schedule for Affective Disorders and Schizophrenia for School-Aged Children-Present and Life Time Version, *LDSQ* Learning Disability Screening Questionnaire, *MOSSF-12*Medical Outcomes Study – Short Form, *MINI v6* Mini International Neuropsychiatric Interview v6.0, *NGO* Non-governmental Organisation, *PCL* PTSD checklist, *PDS* Posttraumatic diagnostic scale, *PMDC* Post Migration Difficulties Checklist, PMLD – Post-migration living difficulties and detention experiences checklist, *POMS* Profile of Mood States, *PSWQ* Penn State Worry Questionnaire, *PTSDRI* Posttraumatic Stress Disorder Reaction Index, *QOLI* Quality of Life Inventory, *R-IES-13* Revised Impact of Events Scale-13, *SAPAS* Standardized Assessment of Personality Abbreviated Scale, *SCAS* Spence Children’s Anxiety Scale, *SCID-IV* Structured Clinical Interview for DSM-IV Axis I Disorders, *SCL-90-R* Symptoms Checklist-90-R, *SDQ* Strengths and Difficulties Questionnaire, *SIS* Suicide Ideation Scale, *T-FAST* Truncated Firestone Assessment of Self destructive Thoughts, *TSCYC-SP* Trauma Symptom Checklist for Young Children – Spanish Version, *UCLA PTSD-RI* UCLA Posttraumatic Stress Disorder Reaction Index, *WHOQOL-Brief* World Health Organisation Quality of Life Short Version

**Table 2:** Quality Review of 26 studies

Quality Question 1-7							
Study	Clear focus on question	Appropriate design	Appropriate sampling method	Appropriate sample	Tolerable level of non-participation	Exposure (detention) appropriately addressed	Outcomes (mental health symptoms and disorders) appropriately addressed
Brabeck & Xu [59]	High	High	High	Intermediate	Intermediate	Intermediate	Low
Cleveland & Rousseau [38]	High	High	Intermediate	High	Intermediate	High	High
Coffey et al. [40]	High	High	Intermediate	Intermediate	Intermediate	High	High
Cohen [50]	Intermediate	Low	Low	Low	Low	Intermediate	Intermediate
Cwikel et al. [37]	High	High	Low	Intermediate	Low	High	High
Graf et al. [47]	High	High	High	Intermediate	Low	High	High
Green & Eagar [36]	High	Intermediate	High	Intermediate	High	High	Intermediate
Hedrick [49]	High	High	High	Intermediate	High	Intermediate	Intermediate
Ichikawa et al. [41]	High	High	High	High	High	High	High
Keller et al. [44]	Intermediate	High	Intermediate	High	High	High	High
Lorek et al. [52]	High	High	Intermediate	Intermediate	Low	High	Intermediate
Mares [54]	High	Intermediate	Intermediate	Intermediate	Low	High	High
Mares & Jureidini [53]	High	High	Intermediate	Intermediate	Intermediate	High	Intermediate
Momartin et al. [51]	High	High	Intermediate	Intermediate	High	High	High
Puthoopparambil et al. (2009)	High	High	High	Intermediate	Intermediate	High	High
Robjant et al. [11]	High	High	Intermediate	High	High	High	High
Rojas-Flores, L. [57]	High	High	Intermediate	Intermediate	Low	Intermediate	High
Rothe et al. [55]	Intermediate	High	Intermediate	Low	Low	High	Intermediate
Rothe et al. [56]	High	High	Low	Low	Low	High	High
Sen et al. [45]	High	High	Intermediate	Intermediate	Intermediate	High	High
Sobhanian et al. [42]	High	Intermediate	Low	Intermediate	Low	High	High
Steel et al. [23]	High	High	High	Intermediate	High	High	Intermediate
Steel et al. [43]	High	High	High	Intermediate	High	High	High
Steel et al. (2011)	High	High	High	High	High	High	High
Young & Gordon [48]	High	High	High	Intermediate	Intermediate	Intermediate	High
Zwi et al. [58]	High	High	Intermediate	High	Intermediate	High	High
Quality Question 8-13							
Study	Consideration of cofounders	Appropriate conduction of statistical analyses	Reporting of confidence intervals	Consideration of ethical issues	Adequate support for conclusions	Generalisability of findings	Total Score
Brabeck & Xu [59]	High	High	High	Intermediate	High	Intermediate	19/26
Cleveland & Rousseau [38]	High	High	High	High	High	High	24/26
Coffey et al. [40]	Low	High	High	High	High	Intermediate	20/26
Cohen [50]	Low	Low	Low	Intermediate	High	Intermediate	7/26
Cwikel et al. [37]	High	High	Intermediate	Intermediate	High	Intermediate	18/26
Graf et al. [47]	Low	High	Low	High	High	High	19/26
Green & Eagar [36]	High	High	High	Intermediate	High	High	22/26
Hedrick [49]	Intermediate	High	Low	Intermediate	High	High	19/26
Ichikawa et al. [41]	High	High	High	High	High	Intermediate	25/26
Keller et al. [44]	High	High	High	High	High	High	23/26
Lorek et al. [52]	Intermediate	Low	Low	Intermediate	High	Intermediate	14/26
Mares [54]	Low	High	Low	High	High	Intermediate	16/26
Mares & Jureidini [53]	Low	Intermediate	Low	Low	High	Intermediate	14/26
Momartin et al. [51]	High	High	High	Intermediate	High	Intermediate	22/26
Puthoopparambil et al. [46]	High	High	High	High	High	High	24/26
Robjant et al. [11]	High	High	High	High	High	High	25/26
Rojas-Flores, L. [57]	High	High	Intermediate	Intermediate	High	Intermediate	18/26
Rothe et al. (2002a)	Low	Low	Low	Low	High	Low	9/26
Rothe et al. (2002b)	High	High	Low	Intermediate	High	Low	15/26
Sen et al. [45]	Intermediate	High	Low	High	High	High	19/26
Sobhanian et al. [42]	High	High	Low	Intermediate	High	High	15/26
Steel et al. [23]	Intermediate	Intermediate	Low	High	High	Intermediate	19/26
Steel et al. [43]	High	High	Intermediate	High	High	Intermediate	23/26
Steel et al. [39]	Intermediate	Intermediate	High	High	High	Intermediate	24/26
Young & Gordon [48]	Low	High	Low	Intermediate	High	Intermediate	17/26
Zwi et al. [58]	Intermediate	High	High	Intermediate	High	High	22/26

Studies that did not report specifically on people held in immigration detention or removal centres (i.e. facilities detaining foreign national citizens for administrative purposes under immigration powers) were excluded. Studies that did not examine the impact of detention on mental health specifically were also excluded. Therefore any studies examining multi-morbidity involving both mental health disorders and physical disorders (e.g. infectious diseases) were excluded, as were studies focused on the attitudes and experiences of health professionals working within immigration detention. Reviews, grey literature, commentaries, conference abstracts, letters, editorials, books and short surveys were also excluded. We have however referred to relevant grey literature in the introduction and discussion sections.

Studies concerning child and adult detainees have been presented separately for the sake of clarity.

### Search strategy

Relevant studies were identified through electronic searches of Embase (1980 to 2018 week 22), Ovid MEDLINE (1946 to May week 3 2018) and PsycINFO (1806 to May week 3 2018). Searches were limited to English language only. No other restrictions were applied to the searches. All search terms related to the following two main areas: mental health and immigration detention. The full electronic search strategy is presented in Additional file 1.

Electronic searches were supplemented by screening of reference lists of all included primary studies. In addition, the following journals published between 2013 and 2018 were hand searched: Forced Migrant Review, International Migration Review, International Migration, Journal of Refugee Studies, Journal of Traumatic Stress, Refuge, and Torture. Six International experts working in the field of forced migration and health (i.e. psychiatry, psychology and criminology) were contacted to nominate further papers eligible for inclusion. One response was received.

### Data extraction and quality appraisal

Three reviewers (CM, RS, ZC) were involved in the process of independently screening titles and available abstracts of identified articles. If it was unclear whether a reference was relevant for the review, texts were retrieved in full. The same team of reviewers (CM, RS, ZC) independently assessed full texts to verify eligibility based on the above selection criteria. Disagreement was resolved either by discussion or with assistance of a third reviewer (CK, KR or MW). Data from all included studies was double extracted by a team of four reviewers (CM, MW, RS, ZC) into a standardised electronic spreadsheet. The following information was extracted from each study: first author, year of publication, population sampled, country where study was carried out, study design, measures used to assess mental health or psychological domains of quality of life, outcomes and length of immigration detention. The quality of studies which met the inclusion criteria was appraised independently by two from a team of three reviewers (CK, CM, MW) using a modified version of the qualitative Critical Appraisal Skills Programme Checklist [35].

Each item was rated as high (2), intermediate (1) or low (0) quality, resulting in a maximum potential total score of 26 (see Table 2 for breakdown of scores). Discrepancies in scores between reviewers were resolved by discussion.

## Results

**Fig. 1:**
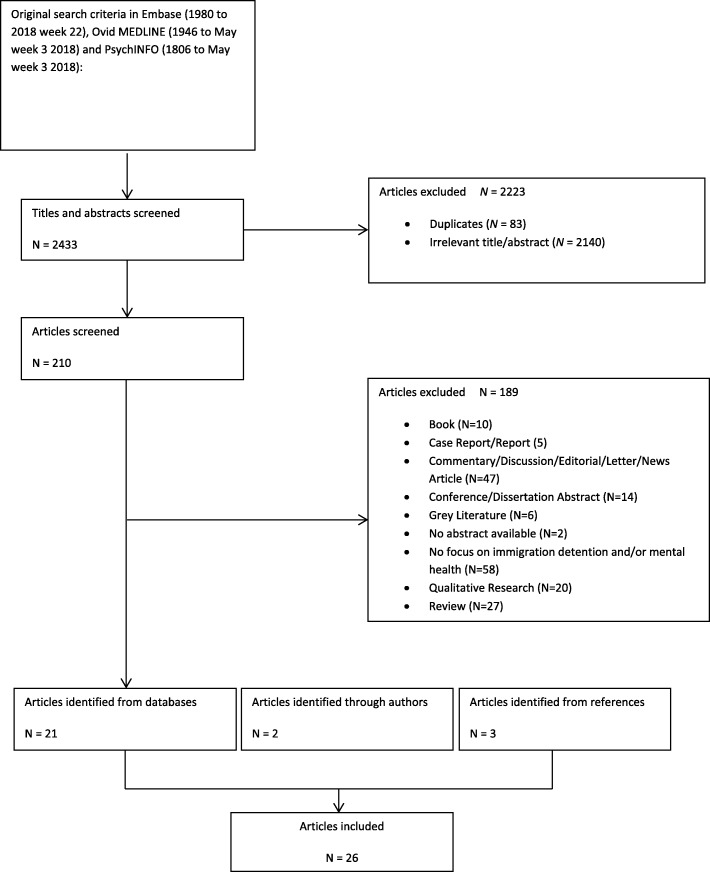
Study flow chart

The study selection process is outlined in Fig. 1. Twenty-six relevant studies reporting on a total of 2099 participants were included in the review. Of these, 16 studies reported on adults, 9 studies on children and families and one reported on both. Studies were conducted in Australia, Canada, Israel, Japan, Sweden, Switzerland, the UK and the US. Not all studies listed individual countries of origin, yet as many as 58 countries were represented within a single study [36]. Key characteristics of all included studies can be found in Table 2.

### Adults

A total of 17 studies reported data on the mental health consequences of immigration detention in adults. These studies involved a total of 1168 participants (Note: this number only includes studies in which primary data was collected). Three studies on parental health are discussed separately in the ‘Child and Families’ subsection below. Six studies were conducted as comparison studies, with a total of 331 non-detained controls. Ten studies were conducted during detention, six were conducted following detention, and one included both current and past detainees. Reasons given for detention varied widely and were not consistently reported. Many were asylum seekers and some studies also included foreign national prisoners or ex-prisoners awaiting deportation, illegal workers and undocumented entrants. Participant ages ranged from 15 to 80 years of age. Approximately two thirds of all participants were male. One study focused exclusively on females, who were illegal sex workers detained in Israel and awaiting deportation [37]. Studies differed widely in the participants’ duration of detention. Some studies reported average duration in terms of the mean, others reported median durations. For those reporting mean durations, these ranged between 4 days to 3.2 years. Reported median durations ranged between 6 to 8 months. Reported ranges fell between 2- to 4 months. Various pre-migratory traumatic events were reported, including but not limited to torture. Where reported, subjection to torture ranged from 12.2–100%.

Overall, 11 studies employed self-report measures, one used self-report measures along with clinical assessment, one used clinical assessments only and four analysed health records. These studies are reviewed below in terms of method of measuring mental health outcomes (self-report and clinical assessments), use of a control design, detention duration and release, and gender.

#### Self-report (see Table 3)

Self-report data (predominantly using clinically validated rating scales) relating to detention experiences consistently identify severe levels of mental health problems amongst detainees. Most studies (*N* = 9) reported on symptoms of anxiety, depression and PTSD, which appear to be the most common forms of mental health difficulties experienced by detainees both during [36–39] and following detention (see below; [40–43]. Whilst not differentiating between types of traumatic experiences, greater trauma exposure prior to detention was significantly associated with higher rates of such symptoms [37, 38, 41]. Gaining temporary protection rather than permanent protection was an independent contributor to the risk of on-going depression and PTSD [43]. Isolation from family members and living alone were also associated with higher rates of such symptoms [41, 43].

**Table 3:** Assessment and Prevalence Rates of Mental Health Difficulties in currently detained and formerly detained Adults

Mental Health Difficulty	Cleveland & Rousseau [38]	Coffey et al. [40]	Cwikel et al. [37]	Ichikawa et al. [41]	Keller et al. [44]	Robjant et al. [11]	Sen et al. [45]	Steel et al. [43]	Steel et al. [39]
Depression	√	88%	79%	√	86%	√	52.5%	√	√
Anxiety	√	–	–	√	77%	72%	12%	√	√
PTSD	√	70%	17%	√	50%	76%	20.8%	√	√
Specific Phobia	–	–	–	–	–				–
Psychotic or Depressive Affective Disorder	–	–	–	–	–	–	10%	–	–
Personality Disorder	–	–	–	–	–	–	34.7%	–	–
Suicidal Ideation	–	–	47%	–	26%	–	–	–	–
Suicide Attempt	–	–	19%	–	3%	–	–	–	–
Somatisation	–	–	40-60%	–	–	–	–	–	–
Autism	–	–	–	–	–	–	15%	–	–
ADHD	–	–	–	–	–	–	14%	–	–
OCD	–	–	–	–	–	–	9%	–	–

Somatization, suicidal ideation, substance use, personality disorder and neurodevelopment disorders were also reported in some studies [37, 44, 45].

Three studies measured the quality of life of detainees and uniformly reported low quality of life ratings, particularly across psychological domains [40, 42, 46]. One of these studies found that levels of quality of life were mediated by the level of perceived support and satisfaction with the care received from detention staff [46].

#### Clinical assessments

Two of the identified studies involved diagnostic clinical assessments. Both of these were restricted to male detainees. Graf and colleagues [47], using the WHO Composite International Diagnostic Interview (CIDI), found a 76% point prevalence of mental health disorders in detainees, with 26.2% meeting criteria for serious mental illness within 4 days of admission. The most common disorders were found to be affective disorders (36%), anxiety disorders (34%) and PTSD (23%). Similar prevalence rates were found in a study by Sen et al. [45], which combined self-report measures with clinical assessments. They found the prevalence of any mental health, developmental or personality disorder to be 74.3%, with over half of the sample screened positive for more than one disorder. Seventy-three percent of the sample reported unmet psychological needs.

#### Secondary health data

Four studies analysed pre-existing health data obtained from detention centres. Data recording was poor across most studies, with authors facing flawed and unsystematic documentation. One study drew on data obtained from health records within detention centres [36]. Despite the reported lack of available records spanning the entire detention period of many detainees, the findings suggest that mental health issues were identified in 6% of detainees. The reason for being detained was also found to contribute to new mental health problems, with unauthorised boat arrivals displaying the highest rates of psychiatric morbidity (27%). Asylum seekers had more health problems including mental health problems than other detainees. Only 7% of all recorded detention centre interventions included formal mental state examinations. A further study reported much higher numbers, with approximately half the detainees suffering from PTSD symptoms [48]. Two studies looked at rates of suicide and/or self-harm using data extracted from incident reports within detention centres in Australia [49] and the UK [50]. Self-harm was estimated to occur in 22% (including attempted hanging) and 12.8% respectively. Where provided, precipitating factors to self-harm revolved around detention conditions and procedural related factors (such as duration of claims) and negative decisions [49]. Suicides in immigration detention were estimated at 222 per 100,000 between 2002 and 2004, with the majority of deaths being caused by hanging [50]. Of the deaths in detainees reported in coroner’s reports and Prisoner’s Ombudsman’s reports, the majority had a history of mental health difficulties including depression, psychosis and PTSD. Overall however, these figures are likely to reflect only the most severe cases in which the mental health difficulties were sufficiently prominent to be identified and recorded by detention staff. Moreover, they are likely to represent the perspective of detention staff rather than of asylum seekers themselves.

#### Comparison studies

Six studies compared the self-reported levels of PTSD, depression and anxiety of detainees to those in a comparison group of non-detained refugees or migrants from a similar background. All six studies showed higher symptom scores and rates of meeting clinical threshold for mental health disorders in the detained groups. This held true when comparing detained asylum seekers with non-detained asylum seekers [38, 41] detained asylum seekers and detained former prisoners with non-detained asylum seekers [11], detained sex workers with non-detained sex workers [37] and detained temporary protection visa holders with non-detained permanent protection visa holders [39, 43, 51]. All but one study [11] found no significant difference in levels of pre-migration trauma exposure between the groups, suggesting that detention contributed independently to PTSD over and above the impact of past traumatic experiences. However, whilst the number of traumatic events may not have differed across groups, the frequency, severity and context of those events were often not assessed.

Whilst no direct comparison between detainees and prison populations have been conducted to date, one study compared findings obtained from one immigration removal centre in the UK against results from prison-based studies utilising the same screening tools [45]. Despite methodological shortcomings and difficulties in making direct comparisons, prevalence rates of depression and PTSD were higher among immigration detainees than in prison populations. Levels of suicidal ideation were high in both groups.

#### Detention duration and release

Eight studies reported on the relationship between length of detention or release from detention and mental health and/or quality of life. Four studies conducted in Australia and the USA found a significant positive correlation between detention duration and symptomatology [36, 43, 44, 48], suggesting that psychological functioning deteriorates with prolonged detention. A UK study found an interaction effect between exposure to interpersonal trauma and length of detention, suggesting that experience of interpersonal trauma and prolonged time in detention resulted in higher anxiety and depression scores [11] . The authors suggest that the interaction they found may also reflect the complex relationship between stage of claim and detention duration within the UK. However, even brief detention (median 17.5 days) has been found to have negative mental health consequences [38].

In contrast, two studies did not find a statistically significant correlation between detention duration and quality of life [42, 46], although a decreasing trend was found in one of these studies [46]. Here, the small sample size as well as the use of quality of life scales rather than mental symptom measures may have resulted in reduced sensitivity to the effects of detention. Furthermore, one of the two ‘negative’ studies indicated a significant increase of suicidal ideation with detention duration [42].

One study employed a longitudinal design with a two-month follow up, indicating not only that detention exacerbates psychological symptoms over time, but also that these symptoms were markedly reduced in those participants who had been released prior to the follow-up rating [44]. However, another longitudinal study suggests that symptomatology and social isolation remained higher in former detainees following release when compared to their non-detained counterparts [39].

Three studies conducting single assessments following release from detention suggest that symptoms of depression, anxiety and PTSD endure beyond the detention period, and persist at 10 months [41], 3 years [43] or almost 4 years [40] after release. In the two latter studies, numerous anxiety-related symptoms, such as avoidance of related triggers, nightmares and flashbacks were linked directly to the detention experience. The severity of such long-term impacts, along with continuing sadness, hopelessness, and anger were again found to correlate with detention duration [43].

Despite this, an improvement in quality of life following release was found when asking former detainees to give retrospective ratings of their quality of life during detention and current ratings following release from detention [42]. After release, participants exhibited more satisfaction, improved mood and a decline in suicidal ideation.

#### Gender

To date, an overwhelming proportion of research focuses on males in detention. In those studies in which female detainees were also included, analysis by gender was usually not conducted. In one study, females were deliberately excluded from the sample due to differing experiences in comparison to men [41].

Only two studies specifically examined gender differences, with one finding no significant differences between genders [38] and the other reporting higher rates of trauma/PTSD in females [48]. Female detainees were also found to be more vulnerable to the effects of more prolonged detention when compared to males.

One study concentrated exclusively on female sex workers in detention, finding high rates of substance abuse, depression, somatic symptoms and PTSD amongst the women [37]. However, this sample of Russian sex workers in Israel differs substantially from other groups of detainees, complicating the generalizability and comparability of these findings. The mental health consequences of detention for female detainees specifically therefore remain unknown based on the studies reviewed.

### Children and families

Ten studies reported data on the mental health of children and/or families within the context of detention. This review reports on 629 children and young people (across 9 studies) and 302 parents (across 5 studies). Two studies were conducted as comparison studies, with a total of 96 controls. Seven studies were conducted while participants were held in detention, and three were conducted following detention. Again, detention duration was differentially reported. Where reported, the means fell between 6 months and 2 years and 4 months and the ranges spanned from 11 days to 2 years and 8 months across studies. The ages of children spanned from childhood to adolescence, ranging from 11 months – 19 years. Both male and female children were represented, yet gender distributions were largely unreported.

Overall, 5 studies used either self-report or parent/teacher report measures Two studies combined clinical assessment with self-report measures to evaluate psychiatric vulnerability [23, 52]. This allowed not only for an estimation of prevalence rates of psychiatric disorders, but also for retrospective accounts of mental health difficulties preceding detention. One study reported clinical outcomes of referrals into Child and Adolescent Mental Health Services (CAMHS) from a detention centre [53]. Two further studies reported on data collected as part of an Australian Human Rights Commission Data Set [48, 54].

A wide range of psychological disturbances mirroring and extending those of adults was found across all three studies which also all included clinical assessments (see Table 4). All children evidenced at least one psychiatric disorder, most frequently depression, anxiety, PTSD and somatization, depending on the diagnostic categories used. The overwhelming majority also struggled with sleeping (65–100%) and eating problems (100%), suicidal ideation (50%), and self-harm (25–80%). Drawing on data from telephone-administered psychiatric interviews and self-report measures from a near-complete sample of detained families of unspecified ethnicity, Steel et al. [23] found psychiatric disorders to be ten times more likely to occur subsequent to detention when compared to prior to detention. Although based on retrospective reports, such difficulties appeared to be specifically linked to detention experiences. Combining clinical assessments with parent-report and, where possible, triangulating these with self-report measures of mental health in a small sample of child and adolescent detainees, Lorek et al. [52] further identified age-related differences in mental health difficulties. Specifically, whilst elevated levels of emotional and behavioural problems and somatic complaints were common across all age groups, developmental delay and regression were more apparent in younger detainees (aged 3–6). These included frequent crying and withdrawal (100%), disturbed sleep routines (100%), language delays (50%,), loss of previously acquired cognitive skills (33%) return to nappies (38%), enuresis (13%) and encopresis (13%). Amongst older children (aged 7–11), self-report questionnaires suggested high levels of depression (100% not within normal range, 50% above the cut off for clinical depression), anxiety (66% above cut off for clinical anxiety) and PTSD symptomatology (17% approaching clinical cut off) [52]. Though this study did not have a control group, the reported lack of pre-existing mental health problems again suggests that sudden mental health deterioration may be attributable to detention experiences.

**Table 4:** Prevalence Rates of Mental Health Difficulties in Children and Families

	Steel et al. [23] *Diagnostic Interview*		Mares & Jureidini [53] *Diagnostic Interview*		Lorek et al. [52] *Diagnostic Interviews, Parent-report, Self-report*	
	Prevalence Rates in Children (*N* = 20)	Prevalence Rates in Parents (*N* = 14)	Prevalence Rates in Children (N = 20)	Prevalence Rates in Parents (N = 16)	Prevalence Rates in Children (N = 11)	Prevalence Rates in Parents (N = 9)
Mental Health Difficulty						
Major Depressive Disorder	95%	100%	100%	87%	100%	100%
Post-traumatic Stress Disorder	50%	86%	100%	56%	17%	55%
Anxiety Disorder	50% (separation anxiety)	100%	70%	–	36-100%	100%
Sleep Related Difficulties	65%	–	100%	–	91%	–
Eating Related Difficulties	–	–	–	–	100%	–
Oppositional Defiant Disorder / Conduct Problems	45%	–	30%	–	55%	–
Psychotic Symptoms	–	14%	–	25%	–	–
Suicidal Ideation	+ 50%	93%	–	–	–	100%
Self-harm	25%	36%	80%	31%	–	–
Somatic Complaints	–	–	50%	–	91%	–
Hyperactive Behaviour	–	–	–	–	27%	–
Peer Relationship Problems	–	–	–	–	64%	–
Developmental Concerns						
Enuresis	50% of children in middle childhood	n.a.	30% of children aged 6-17	–	13% of children aged 1-4	n.a.
Encopresis	–	n.a.	–	–	13% of children aged 1-4	n.a.
Food Refusal	–	n.a.	–	–	38% of children aged 1-4	n.a.
Return to Nappies	–	n.a	–	–	38% of children aged 1-4	n.a.
Language Regression	–	n.a.	50% of children aged under 5	–	50% of children aged 1-4	n.a.
Attachment Problems	–	n.a.	30% of children aged under 5	n.a.	–	n.a.

Similar developmental differences in psychiatric morbidity were also found in the study of CAMHS assessments of 10 detained families [53]. Developmental concerns such as language regression (50%), attachment problems (30%) and enuresis (30%) were reported in children under 5 years of age, whereas symptoms of PTSD (100% above clinical cut off), depression with suicidal ideation (100% above clinical cut-off), self-harm (80%) anxiety (70%), and somatic symptoms (50%) were more common in older children (aged 6–17).

Regressive behaviours such as enuresis (45% boys, 48% girls) and encopresis (8% boys, 11% girls) were also self-reported by a group of adolescents referred into an infirmary during their time in detention in one study, alongside the more common symptoms of severe to very severe PTSD (very severe 94% boys, 96% girls; severe 6% boys, 4% girls; Rothe et al., 2002a). Following release from detention, 57% of adolescents within the same detention facility were considered to continue to meet a diagnosis of PTSD [56].

A secondary analysis of an Australian Human Rights Commission data set indicated very high rates of severe psychological distress amongst adolescents, evidenced by severe disorders for 85.7% of adolescents [54]. All met criteria for mixed anxiety and depression. Additionally, parent reports suggested a high probability of psychiatric disorder in 75% of their children, and significant emotional symptoms int 78.6%. Drawing on a different subset of the collected data, it was estimated that a third of detained children had sufficient clinical symptoms to necessitate tertiary outpatient assessments [48].

#### Comparison studies

Two studies compared the mental health of children within the detention context against those outside it; both found elevated psychological symptoms within the detained groups [57, 58]. When comparing children of detained or deported parents with peers whose parents were either legal permanent residents or undocumented yet without prior contact with immigration enforcement, parent reports indicated higher levels of PTSD symptoms and trauma in children of detained or deported parents than the other two groups [57]. Parents and clinicians also rated detained children as scoring more highly on internalising problems and negative moods. However, the grouping of detention with deportation in this study makes it difficult to tease out the specific effects of the experience of detention as against those of removal or deportation. The authors concluded that forced separation from parents due to immigration enforcement may be particularly adverse to child mental health.

Similarly, a study comparing detained children to child asylum seekers in the community found detained children to have significantly impaired social-emotional wellbeing as measured by conduct disorder, emotional problems and hyperactivity than their non-detained counterparts [58]. Interestingly, detained children scored higher on peer relations and pro-social subscales, which may be essential skills for children living in challenging circumstances to have, particularly if their parents are unable to adequately meet their needs.

#### Parental mental health

Parents displayed high rates of psychopathology across the three studies (see Table 3). There was a substantial increase in reported suicidal ideation. Further, whilst in one study all parents reported the ability to care adequately for and support their children prior to detention, only one of 14 parents remained able to do so during detention [23].

One study reported on the effects of actual or threatened detention and deportation on family units using self-report measures [59]. Multiple regression analysis revealed that parents’ legal vulnerability has an impact on the family environment including the quality of the parent-child relationship (27.1% of variance), as well as on overall child well-being (30.6% of variance). In other words, the greater the parents’ legal vulnerability, the greater the reported consequences of detention and deportation. However, the interchangeable use of the terms ‘detention’ and ‘deportation’ again make it difficult to discern the specific effect of detention.

In keeping with reports on children and adolescents, the secondary analyses revealed 83% of parents to have severe disorders [54]. Again, all participants met criteria for mixed anxiety and depression. When asked about concerns surrounding their child’s emotional wellbeing, mental health and development, 67% identified concerns around emotional and mental health (e.g. nightmares, worry, sadness) and 23% identified concerns around development (e.g. poor eating and low weight).

#### Detention duration and release

Four studies investigated the relationship between detention duration and mental health difficulties. One study found that at 12-month follow up, the mental health of families who remained in detention further deteriorated during incarceration [53]. The three remaining studies did not find a significant correlation between length of detention and mental health problems [54, 56, 58]. However, the authors concluded that this might be due to small sample sizes, the lack of available data on detention duration as well as the possibility of a ceiling effect demonstrating extensive mental health difficulties.

## Discussion

### Key findings of this review

Adverse mental health consequences of immigration detention are consistently recognised across the literature. Such findings prevail even in countries where detention standards are regarded as relatively benign [46, 52]. Much of the clinical literature reports high levels of anxiety, depression and PTSD and poor quality of life. Whilst such mental health difficulties cannot be viewed in isolation from past histories and pre-detention traumas, which are not consistently measured across studies, controlled studies with non-detained controls uniformly suggest greater symptoms and ‘caseness’ (i.e. meeting diagnostic criteria for a specific mental health condition) in detained samples. This indicates that detention plays an independent role in contributing to poor mental health outcomes amongst asylum seekers. Nonetheless, being an asylum seeker or having greater trauma exposure of any kind (whether torture or other exposure) prior to detention seems to be associated with higher rates of anxiety, depression and PTSD in the context of such detention (e.g. [41]). Similarly, greater isolation from families and living alone has been found to increase such symptoms in immigration detainees (e.g. [43]).

All adult studies examining the association between detention duration and mental health severity (*N* = 5) demonstrate a significant relationship between detention duration and mental health deterioration. No such correlation was found for length of detention and quality of life (*N* = 2), although a negative trend was found in one of these studies [46]. Additionally, perceived support from detention staff was found to impact positively on quality of life, highlighting the importance of staff behaviour on the wellbeing of detainees but not necessarily having any effect on their mental health problems. Symptom severity has been found to decrease and quality of life to increase following release, but mental health difficulties persist well beyond release. Despite the prominence of mental health disorders amongst detainees, significant barriers to the identification and treatment of those needs remain [47].

The comparatively smaller body of research on detained children and families (*N* = 10 and a total of 629 children studied) echoes the findings in adults summarised above. It should be noted however that the detention of children is fairly uncommon across several countries including the UK, yet remains relatively widely practiced in some European countries [60] and, as recently widely publicised, in the USA. Across those settings and jurisdictions where it remains permitted, profound and far-reaching mental health difficulties are found amongst detained children and young people. Only one in four studies found the severity of mental health difficulties to increase with time the children spent in detention [53]. The other three studies found no correlation between detention duration and mental health. While this has not been researched, this may be explained by the possibility that even small durations of detention are traumatic and harmful to children. The nature of the mental health difficulties found differs across age groups; whereas younger children display developmental regression and primarily externalising behaviours, symptoms of depression, anxiety and PTSD are prominent in older children. Psychosomatic symptoms or somatization appear to be common across developmental stages. This may reflect the difference in experiencing and communicating psychological difficulties in children and young people [61, 62], but may also be understood as a reflection of the somatic component within the symptom pattern of complex PTSD [63]. Whilst detention itself may compromise child development and psychosocial health, as well as aggravating existing trauma, it may also lead to a breakdown of family units. The detention environment may therefore undermine adequate parenting abilities and leave parents feeling disempowered, as they struggle with their own mental health difficulties. Taken together, this may prevent parents from being able to provide support necessary for healthy child development, leaving children at risk of adverse mental health outcomes [64]. It remains unclear however, whether detaining children together with their parents or forcibly separating children from their families is more harmful to the children’s mental health. Further, it is currently unknown how rates of parental mental health difficulties compare to those of single adults and those whose families are not detained. Finally, there is a dearth of quantitative research on the mental health of unaccompanied minors who are detained. With the current number of unaccompanied minors rising across the globe, research into the effects of immigration detention on this subgroup of children is of high importance, particularly with view to the possibility that some unaccompanied minors may be inappropriately treated as though they were adults, consequently increasing their risk of exploitation and harm [65].

Overall, the findings suggest that detention exacerbates the mental health burden of asylum seekers and refugees and that such detention should be viewed as a traumatic experience in and of itself. This may be particularly true for those detainees who are particularly vulnerable prior to detention. Individuals with pre-existing mental health difficulties may also be less effective self-advocates and therefore more likely to be detained [11], despite recommendations and policies to the contrary [31].

### Key strengths and limitations of this review

A comprehensive search strategy, as well as independent screening, data extraction and quality appraisals were applied to ensure a systematic approach to synthesising the literature on the mental health consequences of immigration detention. Adherence to the PRISMA guidelines further increased the methodological rigour and clarity of the review. We sought to ensure that all research that met out criteria was identified through additional contact with expert researchers in the field. Finally, the exclusion of grey literature, as well as confining the search to papers written in English, inevitably resulted in the exclusion of some papers which may have limited the overall understanding of detainee mental health.

#### What this review adds to existing reviews

To the best of our knowledge, the current review is the first systematic review on the mental health of immigration detainees since our previous review published in 2009 [14]. Although excluding grey literature, it includes a larger number of clinical studies than previous reviews [30] and is not limited to detainees with a recorded history of torture [66]. The current review supports findings from the 2009 review and additionally highlights the importance of considering the impact of immigration detention on the mental health of families. It also includes multiple comparison studies which highlight the independent effect of such detention on mental health over and above that of uncertain immigration status. However, it also highlights the dearth (and consequent future importance) of further high quality research directly investigating vulnerability within the immigration detention context.

### Key limitations of the available evidence and their implications for future research

Numerous methodological problems exist at primary study level, which may restrict the conclusions that can be drawn in this review but also indicate where further research is needed. The evidence to date remains sparse due to the multiple obstacles hindering such research. These include restricted access opportunities into detention facilities as well as the prohibiting of random sampling (e.g. [38, 44]). This may have led to an increased reliance on qualitative rather than quantitative methods, particularly in the study of detained children. Such studies have not been included in this review. With regards to studies conducted within detention centres, participants are often recruited through community areas within the detention centres (e.g. [11]), possibly introducing a bias towards those detainees who are most healthy. Recruitment of participants in studies conducted following release from detention, often occurs through lawyers, community organisations or other support services (e.g. [41]). Most studies did not describe the detention regime in sufficient detail to allow for this to be considered as a potential influencing factor. This, together with clearer differentiation between the traumatic effects of detention itself from those of the detention environment) is an important focus for future research. It also carries the implication that future studies should provide as much information as possible about the detention environment in order to facilitate judgment as to the generalisability of the findings. Participation rates are often unknown. It is therefore possible that the recruited samples differed systematically from detainees who did not access community facilities or come into contact with support services. Samples may therefore represent only the most vulnerable individuals in need of support, or conversely, the most highly functioning who are able to access appropriate services. Further research in more representative populations is needed to allow more confident generalisation of the findings to the detainee population as a whole. Additionally, the probability of being detained depends to some extent to sex and nationality, resulting in predominantly male populations of certain national origins [38]. Whilst a broad range of nationalities is represented across studies, a paucity of research focusing on women is evident. Differing nationalities contribute to the vast heterogeneity of samples, along with varying past experiences and traumatic histories, migration statuses, and detention systems and immigration policies within host countries. Correspondingly, language may be an important complicating factor, acting both as a prominent exclusion criterion or a possible cause of misinterpretation or inability to express mental health difficulties clearly [47]. Communicating mental health problems may additionally be compounded by differing expressions of distress and trauma, alongside differing attitudes and experiences of mental health within countries of origin [67]. However, participants from a wide range of cultural backgrounds seem to endure similar psychological ill-health when subjected to detention.

Multiple designs as well as a diversity of methodology and instruments used are evident across studies. Whilst these may differentially contribute to the overall understanding of mental health problems amongst detainees, they also limit comparability. The majority of research is conducted as cross-sectional studies, which warrant caution in the drawing of casual inferences. The suggested causal link between detention and worsening of mental health is however consistent with a limited number of longitudinal studies and comparison studies, implying that detention not only exacerbates existing mental health disorders, but also contributes independently to the onset of new ones. Nonetheless, isolating the effects of detention alone remains a complex task and additional longitudinal research is needed in order to establish the mental health trajectories of detainees, from the very early days of being arrested and detained. This may also include investigating the adverse effects of being detained multiple separate times at different lengths, as well as the effects that experiences in detention may have on people’s ability to navigate and integrate into new societies. Finally, the frequent reliance on a range of self-report questionnaires, which have often not been validated specifically for the use with detainee populations, may be problematic though it is in our view unlikely that this significantly affects the overall validity of the findings. Moreover, measures often focus solely on symptoms of PTSD, depression and anxiety, which may fail to investigate other disorders such as schizophrenia and other non-affective disorders as may be the case in refugees as a whole [5]. Whilst self-report questionnaires are undoubtedly useful for the purpose of screening for mental health problems, outcomes of such self-report measures should not be equated with formal psychiatric diagnoses, which require clinician-administered interviews. To date, there is a limited use of diagnostic clinical assessments to complement self-report measures, and where these are conducted, the samples are restricted to adult male detainees only.

### Conclusions and practical recommendations

Taken as a whole, the body of research reviewed in this paper consistently illustrates severe mental health consequences amongst detainees across a wide range of settings and jurisdictions. In view of this, in terms of provision of care, consideration of mental health disorders and their sequelae should become routine in making the decision whether or not to detain, and in monitoring the welfare of those already in detention. Comprehensive sociocultural assessments should be carried out by care providers with transcultural knowledge, skills and practices [68]. These assessments should lead to adequate treatment for individuals in detention who suffer from mental health difficulties. Additionally, there is a need to identify and address vulnerability within an overall asylum policy and more specifically a detention policy that avoids or minimises further harm being done to vulnerable people. For this, it is important to take into account multiple varying expressions of distress and vulnerability, which should not be reduced to Western conceptualisations. Moreover, a broad concept of past trauma is needed which is not restricted to the definition of torture according to Article 1 of the Convention Against Torture [69], but rather focuses on the severity of harm to which the individual has been subjected [70]. Further, there should be a greater focus on minimizing length of detention. Our findings add weight to the call in the UK and other countries to introduce a statutory limit of duration of detention, such as exists in all other EU countries [71]. The increasing evidence for the negative impact of immigration detention on vulnerable populations is particularly policy-relevant. Similarly, there is insufficient attention paid to child protection and safety, or to adequate education and health protection in countries where children continue to be detained, despite the recognised vulnerability and risk of children and young people and their protection under the UN Convention on the Rights of the Child [72].

## Additional file

Additional file 1: Systematic Review Immigration Detention. (DOCX 29 kb)

## Abbreviations

CAMHS: Child and Adolescent Mental Health Service
EU: European Union
PTSD: Post-traumatic stress disorder
UK: United Kingdon
UNHCR: United Nations High Commissioner for Refugees
USA: United States of America
WHO: World Health Organization
