# Supplementary material for: The impact of immigration detention on mental health: a systematic review
Source: BMC Psychiatry. 2018 Dec 6;18:382. doi: 10.1186/s12888-018-1945-y (PMC6282296; doi:10.1186/s12888-018-1945-y)
Supplement: Supplementary file 1 — Systematic Review Immigration Detention. (DOCX 29 kb) [file 12888_2018_1945_MOESM1_ESM.docx]

Additional file 1: Search Terms full Example List

Systematic Review Immigration Detention

*Search conducted 14/06/2018*

Databases:

Embase (1980 to 2018 week 22), Ovid MEDLINE (1946 to May week 3 2018) and PsychINFO (1806 to May week 3 2018)

 Search terms:

1. (asylum adj1 seek*).mp. [mp=ti, ab, hw, tn, ot, dm, mf, dv, kw, nm, kf, px, rx, an, ui, tc, id, tm]

2. (Asylumseeker* or Asylum-seeker*).mp. [mp=ti, ab, hw, tn, ot, dm, mf, dv, kw, nm, kf, px, rx, an, ui, tc, id, tm]

3. Asylum applicant*.mp. [mp=ti, ab, hw, tn, ot, dm, mf, dv, kw, nm, kf, px, rx, an, ui, tc, id, tm]

4. (Asylum adj1 claim*).mp. [mp=ti, ab, hw, tn, ot, dm, mf, dv, kw, nm, kf, px, rx, an, ui, tc, id, tm]

5. (Refuge* or Migrant* or Immigrant*).mp. [mp=ti, ab, hw, tn, ot, dm, mf, dv, kw, nm, kf, px, rx, an, ui, tc, id, tm]

6. Refugees.mp. [mp=ti, ab, hw, tn, ot, dm, mf, dv, kw, nm, kf, px, rx, an, ui, tc, id, tm]

7. 1 or 2 or 3 or 4 or 5 or 6

8. Detention.mp. [mp=ti, ab, hw, tn, ot, dm, mf, dv, kw, nm, kf, px, rx, an, ui, tc, id, tm]

9. (Depriv* adj2 liberty).mp. [mp=ti, ab, hw, tn, ot, dm, mf, dv, kw, nm, kf, px, rx, an, ui, tc, id, tm]

10. (Detain or Detained).mp. [mp=ti, ab, hw, tn, ot, dm, mf, dv, kw, nm, kf, px, rx, an, ui, tc, id, tm]

11. Imprison*.mp. [mp=ti, ab, hw, tn, ot, dm, mf, dv, kw, nm, kf, px, rx, an, ui, tc, id, tm]

12. Incarcerat*.mp. [mp=ti, ab, hw, tn, ot, dm, mf, dv, kw, nm, kf, px, rx, an, ui, tc, id, tm]

13. (Reception adj1 cent*).mp. [mp=ti, ab, hw, tn, ot, dm, mf, dv, kw, nm, kf, px, rx, an, ui, tc, id, tm]

14. (Asylum adj1 cent*).mp. [mp=ti, ab, hw, tn, ot, dm, mf, dv, kw, nm, kf, px, rx, an, ui, tc, id, tm]

15. (Accomodation adj1 cent*).mp. [mp=ti, ab, hw, tn, ot, dm, mf, dv, kw, nm, kf, px, rx, an, ui, tc, id, tm]

16. Temporary protection.mp. [mp=ti, ab, hw, tn, ot, dm, mf, dv, kw, nm, kf, px, rx, an, ui, tc, id, tm]

17. Custod*.mp. [mp=ti, ab, hw, tn, ot, dm, mf, dv, kw, nm, kf, px, rx, an, ui, tc, id, tm]

18. (Prison* or jail*).mp. [mp=ti, ab, hw, tn, ot, dm, mf, dv, kw, nm, kf, px, rx, an, ui, tc, id, tm]

19. 8 or 9 or 10 or 11 or 12 or 13 or 14 or 15 or 16 or 17 or 18

20. 7 and 19

21. remove duplicates from 20
